# Kinematic Effect on the Navicular Bone with the Use of Rearfoot Varus Wedge

**DOI:** 10.3390/s22030815

**Published:** 2022-01-21

**Authors:** Álvaro Gómez Carrión, Maria de los Ángeles Atín Arratibel, Maria Rosario Morales Lozano, Carlos Martínez Sebastián, Blanca de la Cruz Torres, Rubén Sánchez-Gómez

**Affiliations:** 1 Nursing Department, Faculty of Nursing, Physiotherapy, and Podiatry, Universidad Complutense de Madrid, 28040 Madrid, Spain; alvaroalcore@hotmail.com (Á.G.C.); matin@ucm.es (M.d.l.Á.A.A.); rmorales@ucm.es (M.R.M.L.); carlos_mar_seb@hotmail.com (C.M.S.); 2 Department of Physiotherapy, University of Seville, c/Avicena, s/n, 41009 Seville, Spain; bcruz@us.es

**Keywords:** rearfoot varus wedge, navicular bone, calcaneus bone, midfoot joint, midtarsal joint, foot orthosis, Polhemus device

## Abstract

Background: The rearfoot varus wedge (RVW) is a common treatment for foot pain and valgus deformity. There is research on its effects in the calcaneus, but there is little research on the navicular. More research is needed with the use of RVW due to the relationship that exists between the position of the navicular and the risk of suffering an injury. Objectives: this study sought to understand how RVW can influence the kinematics of the navicular bone, measuring their movement with the 6 SpaceFastrak system. Methods: a total of 60 subjects participated in the study. Two sensors were used to measure the movement of the calcaneus and navicular using RVWs as compared in the barefoot position in a static way. Results: there were statistically significant differences, the use of RVWs caused changes in the navicular bone, with subjects reaching the maximum varus movement with the use of RVW 7 mm of 1.35 ± 2.41° (*p* < 0.001), the maximum plantar movement flexion with the use of RVW 10 mm of 3.93 ± 4.44° (*p* < 0.001). Conclusions: when RVWs were placed under the calcaneus bone, the navicular bone response was in varus movement too; thus, the use of rearfoot varus wedge can influence the movement of the navicular bone.

## 1. Introduction

A flat foot is a biomechanical condition characterized by a low medial arch, increased calcaneus eversion, and abduction of the forefoot [1]. The navicular bone is part of the midtarsal joint, specifically of the talo-navicular joint, and is the most important bone in the height of the arch of the foot [2]. The condition of pronated foot affects the height of the navicular and is related to pathologies among others such as medial tibial stress syndrome [3], patellofemoral pain syndrome [4], tibial posterior dysfunction [5], and plantar fasciitis [6].

Foot orthoses are commonly used for the management of lower limb lesions, the main effect being kinematic change such as reduction of the peak of the rearfoot eversion and the tibial internal rotation [7]. Nester et al. [8] quantified the effect of the foot orthoses with a varus wedge, decreasing the pronation range and peak pronation velocity during the contact phase; they also decreased the total range of motion of the rearfoot. These results were duplicated in the studies by Majumdar et al. [9] and McCulloch et al. [10] in which foot orthoses with a varus wedge obtained a reduction in maximum rearfoot eversion when walking and running. The therapeutic effect of foot orthoses is still controversial today. It is common in the use of pathologies, such as patellofemoral pain syndrome, where they can change ankle joint motion, angular impulses [11], and sagittal plane biomechanics [12] during walking and stair ambulation in individuals. Its use has also been demonstrated in the improvement of medial tibial stress syndrome [13]. A few studies have shown that foot orthoses with varus wedges or valgus wedges could generate kinematic changes in misaligned structures; other studies indicated that such orthoses could generate kinetic changes without altering movement [14,15,16]. Nester et al. [17] found no or barely perceptible kinematics changes in the knee, hip, and pelvis caused by varus wedges. There are also studies related to changes that wedges produced in joints [14,15,16,17,18], but these other researchers consider that their effect of wedges is only kinetic [19,20].

Previous studies have shown that the position of the navicular influences the development of lower limb pathologies and its static study of the navicular drop test has shown the existence of a relationship between the risk of injury or being free of injury [3,4,5,6]. As well as the use of medial rearfoot wedges, its effectiveness has been proven in improving pain [13], and so it is necessary to quantify the effect on the navicular with the use of different thicknesses of RVWs, which are commonly used to better understand the effect of its use in foot orthoses in lower limb injuries. That is why the Polhemus Fastrak Patriot will be used, a tridimensional electromagnetic device that has been used in previous studies [21,22,23]. The device allows the assessment of the movement of the calcaneus and the navicular with high precision [24] with good intra-class correlation coefficients and a high level of accuracy: 8 mm in static relative to sensor position and 0.15° regarding sensor orientation. The % error of the device is set at 1.6% [22]. Based on the interest of research in knowing the therapeutic effect of RVWs on the foot and other injuries, the objective of this study was to determine the tridimensional navicular movements response in individuals wearing three different rearfoot varus wedges placed under the calcaneus bone in a static position to clarify the biomechanics effect. This study hypothesizes that the use of the RVWs with different thicknesses can generate movement in the frontal plane of the navicular bone, specifically creating a movement in varus, explaining how you can reduce the pronator moments of the foot and cause other movements in the sagittal plane and in the horizontal plane.

## 2. Materials and Methods

The ethics committee of the Virgen Macarena Hospital, representing a public institution, reviewed this research. According to the Declaration of Helsinki, ethical and human criteria were followed. Subjects were informed of the need to sign the informed consent.

### 2.1. Design, Sample Size, and Subjects

The Complutense University of Madrid carried out the statistical evaluation to calculate the sample size necessary in the cross-sectional observational study and to be able to detect the differences in the navicular position with rearfoot varus wedges vs. barefoot position. The use of the Polhemus Fastrak [22] obtained data for the abduction of the hallux phalanx, showing an increase of 1.01 ± 0.36° to 1.31 ± 0.46° (*p* < 0.05) after the heel wedge of 3 cm. Significant data were obtained for the valgus of the first metatarsal by adding heel wedge height of 3 cm, specifically 2.15 ± 0.10° (*p* < 0.001). We needed 60 subjects with a statistical power of 80%, β = 20%, α = 0.05, and a confidence interval (CI) = 95%. A total of 85 subjects were added, due to the common loss of subjects around 20%. We reviewed everything according to the criteria of Reporting of Observational Studies in Epidemiology (STROBE) and used aleatory consecutive sampling to obtain the result for the study.

The following inclusion criteria were required for participation in this study: (1) subjects of both sexes over 18 years of age; (2) subjects with neutral foot (0 to +5) as determined by the Foot Posture Index [25]; (3) subjects having had no lesions in the lower limbs in previous 12 months; (4) subjects with shoe sizes between number 39 and number 43, according to the validation of the European Union (1 Paris point = 6.66 mm). (5) subjects have not worn foot orthoses before. Subjects were also excluded if they showed, according to Murphy [26], previous surgery on the lower limb, misalignment in locomotor apparatus, presence of asymmetries, presence of morphologies in the foot, or presence of ligamentous hypermobility.

The origin of the study population was the patients of the Podiatry Department of the San Agustín Hospital in Dos Hermanas (Seville) from September 2018 to July 2021. In writing and verbally, they voluntarily agreed to take part and signed an informed consent document. All subjects were informed of the confidentiality of their data based on current legal regulations (Organic Law 15/99 of 13 December).

### 2.2. Measurement Procedures, Instruments, and Variables

**Figure 1:**
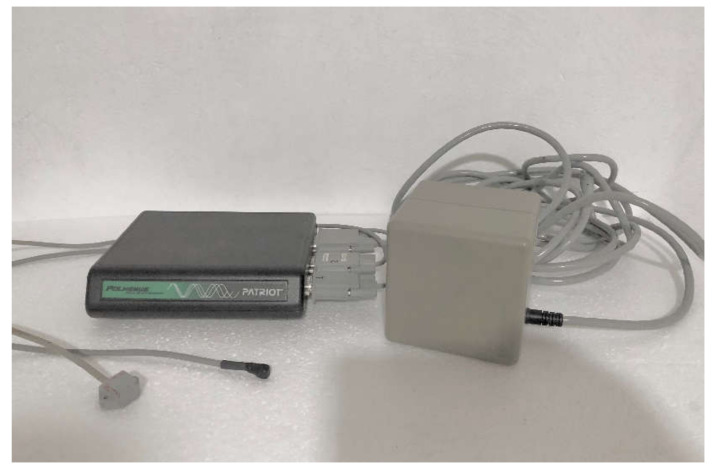
Polhemus system Fastrak. From left to the right: receiver module, two sensors, emitter module.

The mobility measurements of the navicular and the calcaneus bones were carried out with the 6 SpaceFastrak system, which is based on the tracking device (Polhemus Inc., Colchester, VT, USA), configured with a 120 Hz long-range transmitter (Figure 1). This device is based on the emission of a low-frequency electromagnetic field. Within the electromagnetic field generated, it is possible to locate the position and orientation of some sensors that dynamically register their Cartesian coordinates (Z, Y, X) in real-time. The sensor has an accuracy of 0.15° of orientation and 0.8 mm in static, thanks to its integrated system. The area reached between the sensor and the transmitter is 0.76 m and with a reduced precision capacity to 3.05 m. The resolution capacity is 0.5 mm/m position and 0.025° in orientation [22,23,24].

The study subjects were measured in a 6 m long elevated corridor, with the electromagnetic transmitter in the middle of the corridor and raised from the floor to 96 cm. the subjects were asked not to carry metal objects on top that caused distortion of the data. Two electromagnetic sensors were used for this study. One was used as a proximal reference in the calcaneal bone, and another as a distal reference in the scaphoid bone. These sensors can detect the position of the bone in space. A frequency of 60 Hz was used in the sensors.

**Figure 2:**
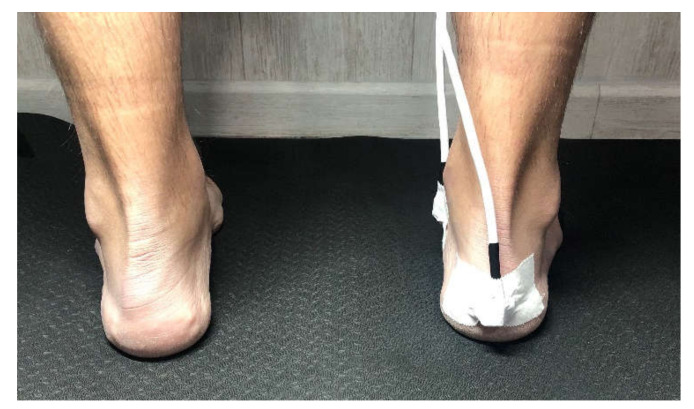
Sensor one. Location placed on the posterior part of the calcaneus.

**Figure 3:**
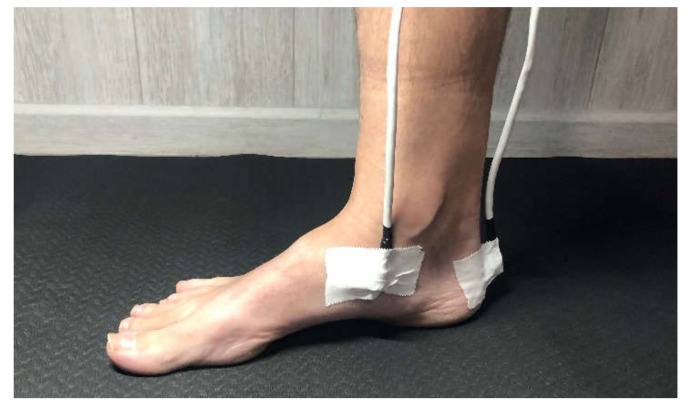
Sensor two. Location placed on the medial navicular tubercle.

The procedure was to place the patient on a stretcher and mark the bone points of interest were marked with a demographic pencil. The patient was then placed in a standing position and the foot posture index was measured. If the subject met the inclusion criteria standing in load, the sensors were fixed to each area marked with double-sided tape and then fixed with Hypafix. The chosen anatomical sites had a minimum of overlying soft tissue. Sensor 1 was placed on the posterior part of the calcaneus (Figure 2) and sensor 2 in a teardrop shape on the navicular tubercle (Figure 3), following the location described by Corwall [24].

For the processing of the data, the special software of the Polhemus system Fastrak is used. The kinematic data were collected thanks to the signals produced by each sensor and were correctly recorded. Before starting to record data, each sensor was calibrated with the subjects close to the electromagnetic transmitter, and at their gait angle with the calcaneus in a relaxed, resting position to establish a reference position. As the “zero” reference value was obtained after calibration and this position was used for subsequent middles [27]. Movement on the medial-lateral axis (X) was defined as dorsal flexion and plantar flexion. Movement on the anterior-posterior axis (Y) was defined as inversion/eversion, and movement on the dorsal-plantar axis (Z) was defined as abduction and adduction.

The measurements were made under the same environmental conditions, taking special care not to have distorting electromagnetic elements nearby, such as electronic devices and metal objects or derivatives, given the negative effects that this would have on data collection according to the manufacturer’s instructions and the field check itself. Three static measurements were made for each varus wedge and barefoot. First, the position of the navicular was measured in barefoot standing; second, the position was measured with the random use of rearfoot varus wedges. This resulted in a total of four measurements, three for the use of wedges (4 mm, 7 mm, and 10 mm) and one barefoot. All tests were performed by placing the same wedge on the contralateral foot so as not to interfere with the patient’s balance during the test or with the difference in the distribution of loads between both feet.

### 2.3. Materials

The wedges were previously made by the researcher, all wedges have the same colour, and were formed of 70 ethylene vinyl acetate (EVA) material with a length of 10 cm and a width of 5 cm. The three wedges had a thickness of 4 mm, 7 mm, and 10 mm. These thicknesses are like those established in the articles reviewed, refs. [8,14,28,29,30].

### 2.4. Variables

The variables that were used to know the spatial movement of the navicular and the calcaneus are summarized in Table 1.

### 2.5. Statistical Methods

The present study was assessed by the statistics unit at the Complutense University of Madrid, which used SPSS Version19.0 (IBM Corp.). An initial Kolmogorov-Smirnov test showed that the data was not normally distributed (*p* < 0.05). *p*-values for multiple comparisons were corrected with a nonparametric paired Friedman test to show that all RVWs variables were different. Bivariate correlations were performed with a Wilcoxon test to determine if there were significant differences between “barefoot” vs. “RVW 4,7,10”.

## 3. Results

**Figure 4:**
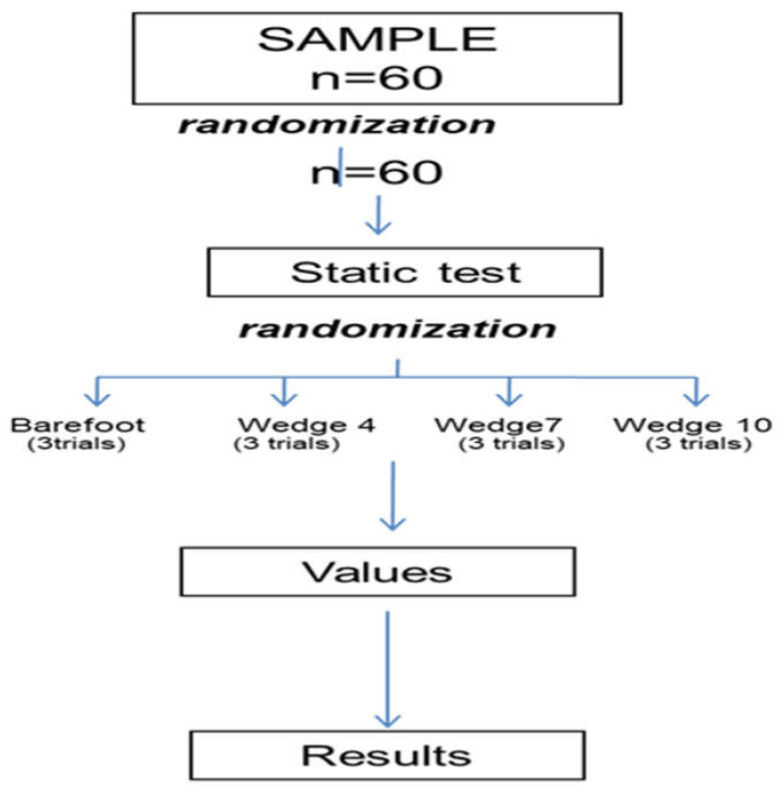
A flowchart about the handling of the different intervention groups.

A total of 85 volunteer subjects were selected initially; a total of 23 of them did not meet the inclusion criteria (presenting diagnosed pathologies, lower limb surgery, genu varus misalignment, genu valgus, genu recurvatum, and genu flexum, presence of dysmetria, etc.), and two subjects were eligible but not recruited for reasons unrelated to the study. A total of 60 subjects (34 women and 26 men) participated in the study; the authors used the baseline condition without wedges as a control group, and it was compared with the result with the use of wedges in the same subjects. (Figure 4) Finally, 60 participants were enrolled.

The sociodemographic characteristics are shown in Table 2. The four physical characteristics measured were homogeneity (age, weight, height, and foot posture index) the applicability of the results was valid for the sample. A normal distribution (*p* > 0.05) was obtained.

### 3.1. Intra-Class Correlation Coefficients, Standard Error of Measurement, and Minimal Change Detectable

The reliability of the data obtained from the two sensors in different under conditions is presented as intra-class correlation coefficients (ICCs) and standard error of measurement (SEM), as shown in Table 3 and Table 4. With the barefoot test having 0.831 as the lowest and with rearfoot varus wedge 7 mm having 0.996 as the highest value for the navicular, and with barefoot test having 0.862 as the lowest and rearfoot varus wedge 7 mm having 0.998 as the highest value for calcaneus, this suggests an “almost perfect” reliability for ICCs. Correlation coefficients (ICCs) were used according to the Landis and Koch specifications to assess the reliability of the measurements: with coefficients less than 0.20, a slight agreement is represented, between 0.21 and 0.40 is fair reliability, between 0.41 and 0.60 is considered moderate reliability, between 0.61 and 0.80 is considered substantial reliability, and between 0.81–1.00 is considered almost perfect reliability. For results of 0.90 or greater, enough reliability is obtained, being a valid average [31]. For SEM, 0.066 for navicular-adduction on barefoot was the lowest and 0.671 was the highest for navicular-plantar flexion with rearfoot varus wedge 10 mm, while 0.045 for calcaneus-valgus with rearfoot varus wedge 7 mm was the lowest and 0.394 was the highest for barefoot. The highest minimal change detectable (MDC) value was 1.861 for navicular-plantar flexion with rearfoot varus wedge 10 mm and the lowest value was 0.240 for navicular-valgus with rearfoot varus wedge 10 mm. For the calcaneus, the highest MDC value was 1.093 for calcaneus-abduction on barefoot and the lowest value was 0.124 for calcaneus-valgus with rearfoot varus wedge 7 mm.

### 3.2. The Navicular Sensor Degrees of Motion

The comparison of the barefoot position and the use of rearfoot wedges on the navicular are summarized in Table 5.

The use of wedges generated significant movement in adduction and abduction, but the mean value of degrees was higher in abduction. With RVW4, the mean range of motion obtained from NAV-ADD is 1.18 ± 1.74° (95% CI 0.73–1.61) (*p* < 0.05) and the mean in NAV-ABD is 1.92 ± 3.24° (95% CI 1.10–2.74) (*p* < 0.05). With the RVW7, the mean range of motion obtained from NAV-ADD is 1.57 ± 2.40° (95% CI 0.96–2.17 (*p* < 0.05) and the mean in NAV-ABD is 2.30 ± 3.60° (95% CI 1.40–3.22) (*p* < 0.05). With the RVW10, the mean range of motion obtained from NAV-ADD is 1.96 ± 2.80° (95% CI 1.25–2.66) (*p* < 0.05) and mean in NAV-ABD is 2.64 ± 4.04° (95% CI 1.60–3.70) (*p* < 0.05).

Another statistically significant result was the movement in plantar flexion that caused the use of rearfoot wedges. With RVW4, the mean range of motion obtained from NAV-PF was 2.27 ± 3.20° (95% CI 1.45–3.09) (*p* < 0.05). With RVW7, the mean range of motion obtained from NAV-PF was 3.46 ± 4.21° (95% CI 2.37–4.54) (*p* < 0.001). With the RVW10, the range of motion obtained from NAV-PF was 3.93 ± 4.44° (95% CI 2.81–5.06) (*p* < 0.001).

The last statistically significant data was the varus movement caused by rearfoot wedges.

With RVW4, the mean range of motion obtained from NAV-VR was 0.86 ± 1.32° (95% CI 0.52–1.20) (*p* < 0.001). With RVW7, the mean range of motion obtained from NAV-VR is 1.35 ± 2.41° (95% CI 0.73–1.97) (*p* < 0.001). With the RVW10, the mean range of motion obtained from NAV-VR was 1.30 ± 2.03° (95% CI 0.77–1.81) (*p* < 0.001) and the mean in NAV-VL is 0.81 ± 1.54° (95% CI 0.41–1.20) (*p* < 0.05).

### 3.3. The Calcaneus Sensor Degrees of Motion

The comparison of the barefoot position and the use of rearfoot wedges on the calcaneus are summarized in Table 6.

The use of wedges generated significant movement in adduction, but with RVW10 the movement was significant for both adduction and abduction. With RVW4, the mean range of motion obtained from CALC-ADD was 1.19 ± 1.74° (IC 95% of 0.74–1.7) (*p* < 0.001). With the RVW7, the mean range of motion obtained from CALC-ADD was 1.67 ± 2.40° (IC 95% of 1.07–2.3) (*p* < 0.001). With the RVW10, the mean range of motion obtained from CALC-ADD was 1.83 ± 2.94° (IC 95% of 1.10–2.60) (*p* < 0.05) and the mean in CALC-ABD was 2.13 ± 3.09° (IC 95% of 1.34–2.93) (*p* < 0.05).

Another statistically significant result was the movement in plantar flexion that prompted the use of rearfoot wedges. With RVW4, the mean range of motion obtained from NAV-PF was 1.77 ± 2.39° (IC 95% of 1.17–2.40) (*p* < 0.001). With RVW7, the mean range of motion obtained from NAV-PF was 2.03 ± 2.50° (IC 95% of 1.40–2.70) (*p* < 0.001). With the RVW10, the range of motion obtained from NAV-PF was 3.00 ± 3.60° (IC 95% of 2.07–3.92) (*p* < 0.001).

The last statistically significant data are the varus movements caused by rearfoot wedges. With RVW4, the mean range of motion obtained from CALC-VR was 1.33 ± 1.70° (IC 95% of 0.90–1.77) (*p* < 0.001). With RVW7, the mean range of motion obtained from CALC-VR was 1.74 ± 2.10° (IC 95% of 1.20–2.27) (*p* < 0.001). With the RVW10, the mean range of motion obtained from CALC-VR was 2.11 ± 2.43° (IC 95% of 1.50–2.72) (*p* < 0.001). The rest of the results did not show statistically significant changes.

## 4. Discussion

The goal of the present study was to assess the effects of different rearfoot varus wedges placed under the calcaneus as well as their effects on the calcaneus’s relationship to the navicular bone. In light of the obtained results, the present study demonstrated that the use of the rearfoot varus wedge generated in subjects’ abduction movements for the transverse plane in the navicular bone (*p* < 0.05), plantar flexion movement for the sagittal plane (*p* < 0.001), and varus movement (*p* < 0.001) for the frontal plane. According to Root’s theory [21], the behavior of the midtarsal joint is a double helix motion in which forefoot movements are opposite to the rearfoot, i.e., when the rearfoot is in valgus motion, the navicular should be opposite, that is in varus motion. Surprisingly, in the present study it has been shown that when a rearfoot varus wedge was placed under the calcaneus and this bone showed varus motion, the navicular bone response had varus movement too. This result between the calcaneal and the navicular bones has been observed in the frontal plane, sagittal plane, and more mildly, in the transverse plane.

Patients given foot orthoses with varus wedges have shown improvements in their overpronation pathologies, indicating that the effect can be both kinetic and kinematic through increasing the varying moments or aligning the talus and the calcaneus [30,32,33]. The study by Shih et al. [34] reveals a therapeutic effect not only on the foot but also on the improvement of anterior knee pain with the use of a varying wedge. We must better understand how these treatments act mechanically, which cardinal planes can change, and which movements would be beneficial to incorporate in our clinical practice.

We have not found studies by other authors that compared the use of the rearfoot varus wedge and examined its effects on the navicular movements. Only one study, performed on a cadaver by Blackwood et al. [21] showed that the position of the rearfoot could alter the movement of the forefoot through the midtarsal joint. When the rearfoot is in valgus, the capacity of movement in the sagittal plane in the metatarsals is increased.

For the results of the range of motion of the navicular in plantar flexion in the sagittal plane with the use of varus wedges, several authors [28,35] stated that with the use of rearfoot varus wedges of 5 mm, 6 mm, and 7 mm, the peak of plantar flexion was increased on the ankle; the data provided lack the mean and standard deviation (SD) of the plantar flexion of the ankle for both phases, referring only to an increase in plantar flexion as the thickness of the wedge increases. The use of a small wedge obtained an average of 14° in plantar flexion; the use of a medium wedge obtained an average of 14° in plantar flexion; and the use of a thick wedge obtained an average of 17° in plantar flexion. In accordance with our findings, Lin et al. [28], reported that when the varus rearfoot wedge correction is increased, the plantar flexion of the joint studied also increased.

Regarding the results of the range of motion of the navicular in varus in the frontal plane, Nester et al. [8] showed the anti-pronation orthoses decreased pronation during the midstance phase and the total rearfoot range of motion; anti-pronation orthoses also decreased the initial peak of pronation and velocity during the contact phase of the gait. These results were also shared by Majumbar [9], who observed that for subjects wearing personalized anti-pronate insoles, there was a decrease in the maximum rearfoot eversion of 3.8° while walking and 2.5° while running. Along the same lines, the study of Ahn et al. [30] demonstrated a greater therapeutic effect of using the varus wedge associated to a control of the talus by means of a medial restraint. Significant improvements were obtained in the relaxed calcaneus position in support in both groups (*p* < 0.05]. These effects were also reflected in the Branthwaite [36] study, which demonstrated that the biplanar orthoses significantly reduced (*p* < 0.05) the maximum eversion by an average of 3.1° compared to the condition without template and that the cobra orthoses reduced the maximum eversion by an average of 2.1° compared to the no-orthoses condition. This difference was close to statistical significance (*p* = 0.058). Similar results were also obtained in the study by McCulloch [10], in which subjects walking with the use of a varus wedge orthosis showed a significant reduction in the degree of pronation, as well as an increase in the duration of the meantime of gait support.

In addition, it could be observed that the higher the wedge we used, the more varus the position of the navicular. A study by Nester [8] used a 3 mm orthosis with a 10° varus wedge from the heel to the head of the first goal, together with a 12 mm arch with maximum navicular height. This is similar to the use of the 4 mm varus wedge in our study, where we also obtained a statistically significant varus movement in the navicular (*p* < 0.001).

The reviewed studies did not collect data for the remainder of the movements of the sagittal plane and horizontal plane. In our case, the varus movement was associated with the rest of the planes. The difference between the studies by Nester [8] and Majumbar [9] and our study was that theirs were performed in dynamics and the wedges were full travel and associated with an internal arch, while we used only a rearfoot varus wedge, thus providing a clear and isolated effect without the bias of the effect of other corrections in the foot. Our study was also carried out in static conditions, thus eliminating the possible distorting effect of the dynamics of the step. A study by Ferber [15] included the comparison of the use of Blake’s inverted orthoses at 15° and 25° correction, with the use of standard orthoses with a 4° varus wedge and without the use of orthoses and found no significant changes between the different groups in the tibia-rearfoot joint coupling. Ferber found only improvement in symptoms with the use of the inverted orthosis. These results are shared by Lee [37], who found the use of Blake´s inverted orthosis obtained significant improvements (*p* < 0.001) in the degree and frequency of childhood flatfoot pain after one and three months of treatment. The values of a study by Butler [14] were not significant either with the use of a 6° varus wedge in either eversion movement, eversion peak, or rearfoot eversion velocity, nor were they values significant in a study by Nigg [19], in which the effects of a varus wedge on the modification of total shoe eversion, total foot eversion, and total internal tibial rotation were typically less than 1 degree compared to the barefoot condition and were not statistically significant. Another study by Nester [17] showed that the pronation in the rearfoot was decreased and the reaction force of the ground was shifted laterally with the use of the medial wedge during the counting phase, obtaining a decrease in the cushioning capacity. Nester obtained generally minimal kinematic effects from knee, hip, and pelvic orthoses.

All the conclusions in the previous literature were in line with the results shown in the present study, which demonstrated that RVW can influence the frontal and sagittal planes and, for some subjects, the transverse planes. In our study, we have tried to relate the effect of a proximal varus wedge placed under the calcaneus bone (proximal point) with another more distal area such as the navicular; the use of a varus wedge has a strong effect on the sagittal plane of the navicular, where a marked plantar flexion was obtained. These results are important for the clinician, since the use of the varus wedge must be associated with the use of an internal arch that controls the force of deformation in the sagittal plane. In addition, this is particularly important for daily clinical practice, as it would explain the etiology of the blisters and chafing that many patients suffer in the medial arch when they are treated with plantar orthoses with a varus or similar wedge.

### 4.1. Limitations

The study has a few limitations, including unwanted movements during the fixation of the sensors to the skin or during the change of treatment. Also, the position of a wedge generates some instability, and although adaptation time was factored in before the measurement was taken, it is possible that there were unwanted movements during the capture. The high sensitivity of the instrument, which can give a dispersion of sample data, is also a limitation.

### 4.2. Future Lines of Investigation

Given the direct implication of the cuboid in the behavior of the midtarsal joint, it is considered important to carry out future studies that take this bone into account for the complete interpretation and understanding of said joint.

Likewise, and given that the foot is a dynamic structure, it is considered important to compare the clean results without movement bias obtained in the present work with the foot’s dynamics during human gait in order to get closer to a real understanding of the clinical pathologies of patients.

## 5. Conclusions

It has been shown that the use of rearfoot varus wedge can influence the movement of navicular under static conditions. The wedges are able to generate a varus movement in the navicular and decrease the valgus of the navicular, and are also capable of generating a great movement of plantar flexion and altering the movement of the horizontal plane of the navicular.

## Figures and Tables

**Table 1:** The table represents the Cartesian coordinate axes (X), (Y), and (Z), for the medial-lateral axis the dorsal flexion is a positive value (X+) and the plantar flexion is a negative value (X-), for the anterior-posterior axis the varus is a positive value (Y+) and the valgus is a negative value (Y-) and for the dorsal-plantar axis the adduction is a positive value (Z+) and the abduction is a negative value (Z-).

Navicular		
Medial—lateral axis (X):	NAV-DF	NAV-PF
Anterior—posterior axis(Y):	NAV-VR	NAV-VL
Dorsal—plantar axis(Z):	NAV-ADD	NAV-ABD
**Calcaneus**		
Medial—lateral axis (X):	CAL-DF	CAL-PF
Anterior—posterior axis(Y):	CAL-VR	CAL-VL
Dorsal—plantar axis(Z):	CAL-ADD	CAL-ABD

Abbreviations: NAV-DF = Navicular-dorsal flexion; NAV-PF = Navicular-plantar flexion; NAV-VR = Navicular-varus; NAV-VL = Navicular-valgus; NAV-ADD = Navicular-adduction; NAV-ABD = Navicular-abduction; CAL-DF = Calcaneus-dorsal flexion; CAL-PF = Calcaneus-plantar flexion CAL-VR = Calcaneus-varus; CAL-VL = Calcaneus-valgus; CAL-ADD = Calcaneus-adduction; CAL-ABD = Calcaneus-abduction.

**Table 2:** Anthropometric characteristics of the subjects.

Variable	*n* = 60 Mean ± SD (95% CI)
**Age** (years)	29.52 ± 9.99 (26.99–32.05)
**FPI** (scores)	1.62 ± 1.36 (1.27–1.96)
**Weight** (kg)	66.92 ± 14.57 (63.23–70.60)
**Height** (cm)	167.67 ± 12.63 (164.47–170.08)

Abbreviations: SD = Standard Deviation; CI = confidence interval (with a 95% confidence interval); FPI = foot posture index.

**Table 3:** ICC, SEM & MDC values of the navicular variables in barefoot condition and wearing rearfoot varus wedges.

			BAR			RVW4				RVW7				RVW10		
Variables	SD	ICC	SEM	MDC	SD	ICC	SEM	MDC	SD	ICC	SEM	MDC	SD	ICC	SEM	MDC
		(95% CI)				(95% CI)				(95% CI)				(95% CI)		
**NAV-ADD**	0.779	0.831	0.320	0.887	1.741	0.984	0.222	0.616	2.377	0.988	0.173	0.479	2.808	0.970	0.481	1.335
		(0.740–0.894)				(0.975–0.990)				(0.981–0.992)				(0.955–0.981)		
**NAV-ABD**	1.700	0.850	0.659	1.826	3.244	0.989	0.347	0.964	3.582	0.993	0.193	0.534	4.046	0.991	0.375	1.039
		(0.770–0.905)				(0.982–0.993)				(0.989–0.996)				(0.987–0.995)		
**NAV-PF**	1.340	0.841	0.535	1.483	3.202	0.989	0.334	0.928	4.219	0.996	0.217	0.602	4.444	0.977	0.671	1.861
		(0.754–0.900)				(0.983–0.993)				(0.994–0.998)				(0.965–0.986)		
**NAV-DF**	0.458	0.979	0.066	0.184	2.490	0.988	0.276	0.767	2.918	0.993	0.118	0.326	2.796	0.985	0.341	0.946
		(0.968–0.987)				(0.981–0.992)				(0.989–0.996)				(0.977–0.991)		
**NAV-VR**	0.947	0.985	0.115	0.320	1.321	0.990	0.133	0.370	2.408	0.989	0.144	0.399	2.035	0.995	0.139	0.386
		(0.977–0.991)				(0.984–0.994)				(0.982–0.993)				(0.993–0.997)		
**NAV-VL**	0.385	0.831	0.158	0.439	0.768	0.983	0.100	0.279	1.153	0.983	0.086	0.240	1.543	0.989	0.159	0.441
		(0.741–0.893)				(0.974–0.989)				(0.974–0.989)				(0.984–0.993)		

Abbreviations: SD = Standard Deviation; ICC = Intraclass correlation coefficient; SEM = Standar error of measurement; MDC = Minimal change detectable CI = Confidence Interval; NAV-ADD = Navicular-adduction; NAV-ABD = Navicular-abduction; NAV-PF = Navicular-plantar flexion; NAV-DF = Navicular-dorsal flexion; NAV-VR = Navicular-varus; NAV-VL = Navicular-valgus; BAR. Barefoot; RVW4. Rearfoot varus wedge 4 mm; RVW7. Rearfoot varus wedge 7 mm; RVW10. Rearfoot varus wedge 10 mm.

**Table 4:** ICC, SEM, and MDC values of the calcaneus variables in barefoot condition and wearing rearfoot varus wedges.

			BAR			RVW4				RVW7				RVW10		
Variables	SD	ICC	SEM	MDC	SD	ICC	SEM	MDC	SD	ICC	SEM	MDC	SD	ICC (95% CI)	SEM	MDC
		(95% CI)				(95% CI)				(95% CI)						
**CALC-ADD**	0.307	0.967	0.056	0.155	1.787	0.990	0.181	0.501	1.666	0.971	0.281	0.780	2.948	0.996	0.185	0.513
		(0.948–0.979)				(0.984–0.994)				(0.956–0.982)				(0.994–0.998)		
**CALC-ABD**	1.062	0.862	0.394	1.093	1.817	0.981	0.250	0.692	1.317	0.995	0.096	0.265	3.090	0.996	0.194	0.537
		(0.788–0.913)				(0.971–0.988)				(0.992–0.997)				(0.994–0.998)		
**CALC-FP**	0.900	0.883	0.307	0.852	2.387	0.979	0.349	0.968	2.027	0.983	0.264	0.733	3.592	0.996	0.231	0.640
		(0.821–0.926)				(0.967–0.987)				(0.974–0.989)				(0.994–0.998)		
**CALC-FD**	0.796	0.891	0.263	0.729	2.235	0.998	0.106	0.294	0.793	0.986	0.094	0.260	1.933	0.995	0.143	0.396
		(0.833–0.931)				(0.997–0.999)				(0.979–0.991)				(0.992–0.997)		
**CALC-VR**	0.484	0.896	0.156	0.433	1.691	0.991	0.161	0.446	1.736	0.982	0.235	0.650	2.439	0.974	0.390	1.080
		(0.840–0.934)				(0.984–0.995)				(0.972–0.989)				(0.961–0.984)		
**CALC-VL**	0.900	0.978	0.135	0.374	1.499	0.989	0.156	0.431	1.034	0.998	0.045	0.124	1.987	0.997	0.103	0.286
		(0.965–0.986)				(0.991–0.996)				(0.997–0.999)				(0.996–0.998)		

Abbreviations: SD = Standard Deviation; CI = Confidence Interval; CAL-ADD = Calcaneus-adduction; CAL-ABD = Calcaneus-abduction; CAL-PF = Calcaneus-plantar flexion; CAL-DF = Calcaneus-dorsal flexion; CAL-VR = Calcaneus-varus; CAL-VL = Calcaneus-valgus; BAR. Barefoot; RVW4. Rearfoot varus wedge 4 mm; RVW7. Rearfoot varus wedge 7 mm; RVW10. Rearfoot varus wedge 10 mm.

**Table 5:** The navicular sensor degrees of motion results in barefoot condition and with 4 mm, 7 mm, and 10 mm rearfoot varus wedge.

	BAR	RVW 4 mm	RVWS 7 mm	RVW 10 mm	*p*-Value BAR	*p*-Value BAR	*p*-Value BAR	*p*-Value RVW 4 mm	*p*-Value RVW 4 mm	*p*-Value RVW 7 mm
Variables	mean (degrees)	mean (degrees)	mean (degrees)	mean (degrees)	vs	vs	vs	vs	vs	vs
	± SD (95% CI)	± SD (95% CI)	± SD (95% CI)	± SD (95% CI)	RVW 4 mm	RVW 7 mm	RVW 10 mm	RVW 7 mm	RVW 10 mm	RVW 10 mm
**NAV-ADD**	0.51 ± 0.68	1.17 ± 1.74	1.57 ± 2.40	1.96 ± 2.80						
**NAV-ABD**	(0.33–0.68)	(0.73–1.61)	(0.96–2.17)	(1.25–2.66)	<0.05 *	<0.05 *	<0.05 *	0.147	<0.001 **	<0.05 *
	0.66 ± 1.7	1.92 ± 3.24	2.30 ± 3.60	2.64 ± 4.04						
	(0.30–1.05)	(1.10–2.74)	(1.40–3.22)	(1.60–3.70)	<0.05 *	<0.05 *	<0.05 *	0.17	0.11	0.13
**NAV-FP**	0.68 ± 1.34	2.27 ± 3.20	3.46 ± 4.21	3.93 ± 4.44						
	(0.38–0.98)	(1.45–3.09)	(2.37–4.54)	(2.81–5.06)	<0.05 *	<0.001 **	<0.001 **	<0.001 **	<0.001 **	<0.05 *
**NAV-FD**	0.31 ± 0.46	1.30 ± 2.49	1.40 ± 2.92	1.31 ± 2.80						
	(0.19–0.42)	(0.67–1.93)	(0.65–2.14)	(0.60–2.02)	0.069	0.43	1.16	0.80	1.51	2.57
**NAV-VR**	0.35 ± 0.95	0.86 ± 1.32	1.35 ± 2.41	1.30 ± 2.03						
	(0.11–0.60)	(0.52–1.20)	(0.73–1.97)	(0.77–1.81)	<0.001 **	<0.001 **	<0.001 **	0.17	<0.05 *	0.22
**NAV-VL**	0,.27 ± 0.39	0.49 ± 0.77	0.66 ± 1.20	0.81 ± 1.54						
	(0.18–0.35)	(0.29–0.68)	(0.37–0.96)	(0.41–1.20)	0.26	0.18	<0.05 *	0.25	0.50	1.08

Abbreviations: SD = Standard Deviation; CI = Confidence Interval; NAV-ADD = Navicular-adduction; NAV-ABD = Navicular-abduction; NAV-PF = Navicular-plantar flexion; NAV-DF = Navicular-dorsal flexion; NAV-VR = Navicular-varus; NAV-VL = Navicular-valgus; BAR. Barefoot; RVW4. Rearfoot varus wedge 4 mm; RVW7. Rearfoot varus wedge 7 mm; RVW10. Rearfoot varus wedge 10 mm. *p* value = level of significance; *p* < 0.05 * (with a 95% confidence interval) was considered statistically significant and *p* < 0.001 ** (with a 95% confidence interval) was considered strong statistically significant.

**Table 6:** The calcaneus sensor degrees of motion result in barefoot condition and with 4 mm, 7 mm, and 10 mm rearfoot varus wedge.

	BAR	RVW 4 mm	RVWS 7 mm	RVW 10 mm	*p*-Value BAR	*p*-Value BAR	*p*-Value BAR	*p*-Value RVW 4 mm	*p*-Value RVW 4 mm	*p*-Value RVW 7 mm
Variable	mean (degrees)	mean (degrees)	mean (degrees)	mean (degrees)	vs	vs	vs	vs	vs	vs
	± SD (95% CI)	± SD (95% CI)	± SD (95% CI)	± SD (95% CI)	RVW 4 mm	RVW 7 mm	RVW 10 mm	RVW 7 mm	RVW 10 mm	RVW 10 mm
**CALC-ADD**	0.17 ± 0.68	1.19 ± 1.79	1.67 ± 2.40	1.83 ± 2.94						
	(0.10–0.24)	(0.74–1.7)	(1.07–2.3)	(1.10–2.60)	<0.001 **	<0.001 **	<0.05 *	<0.05 *	0.11	0.6
**CALC-ABD**	0.55 ± 1.06	1.15 ± 1.81	1.32 ± 2.14	2.13 ± 3.09						
	(0.30–0.79)	(0.70–1.62)	(0.77–1.87)	(1.34–2.93)	0.18	0.3	<0.05 *	1.128	<0.05 *	<0.001 **
**CALC-FP**	0.35 ± 0.90	1.77 ± 2.39	2.03 ± 2.50	3.00 ± 3.60						
	(0.15–0.57)	(1.17–2.40)	(1.40–2.70)	(2.07–3.92)	<0.001 **	<0.001 **	<0.001 **	0.189	<0.001 **	<0.001 **
**CALC-FD**	0.37 ± 0.79	0.85 ± 2.23	0.79 ± 1.40	0.95 ± 1.93						
	(0.18–0.56)	(0.27–1.43)	(0.44–1.15)	(0.45–1.45)	1	0.87	1.67	1.05	0.89	0.78
**CALC-VR**	0.20 ± 0.49	1.33 ± 1.70	1.74 ± 2.10	2.11 ± 2.43						
	(0.09–0.31)	(0.90–1.77)	(1.20–2.27)	(1.50–2.72)	<0.001 **	<0.001 **	<0.001 **	0.054	<0.001 **	0.07
**CALC-VL**	0.40 ± 0.90	0.92 ± 1.50	1.03 ± 1.82	1.02 ± 1.98						
	(0.17–0.63)	(0.39–1.07)	(0.56–1.50)	(0.50–1.52)	1.07	0.86	0.48	<0.05 *	0.46	2.17

Abbreviations: SD = Standard Deviation; CI = Confidence Interval; CAL-ADD = Calcaneus-adduction; CAL-ABD = Calcaneus-abduction; CAL-PF = Calcaneus-plantar flexion; CAL-DF = Calcaneus-dorsal flexion; CAL-VR = Calcaneus-varus; CAL-VL = Calcaneus-valgus; BAR. Barefoot; RVW4. Rearfoot varus wedge 4 mm; RVW7. Rearfoot varus wedge 7 mm; RVW10. Rearfoot varus wedge 10 mm. *p* value = level of significance; *p* < 0.05 * (with a 95% confidence interval) was considered statistically significant and *p* < 0.001 ** (with a 95% confidence interval) was considered strong statistically significant.

## Data Availability

Not applicable.
